# 17-Alpha-Hydroxyprogesterone vs. Placebo for Preventing of Recurrent Preterm Birth: A Systematic Review and Meta-Analysis of Randomized Trials

**DOI:** 10.3389/fmed.2021.764855

**Published:** 2021-12-01

**Authors:** Abdulaali R. Almutairi, Hadir I. Aljohani, Nouf S. Al-fadel

**Affiliations:** Drug Sector, Saudi Food and Drug Authority, Riyadh, Saudi Arabia

**Keywords:** 17-alpha-hydroxyprogesterone caproate, 17-OHPC, preterm birth, recurrent preterm birth, systematic review and meta-analysis

## Abstract

**Background:** Preterm birth (PTB) is a leading cause of neonatal morbidity and mortality.

**Objective:** To estimate the effect of 17-alpha-hydroxyprogesterone caproate (17-OHPC) compared to placebo in singleton gestations for reducing the risk of recurrent PTB and neonatal morbidity and mortality.

**Work Design:** Systematic review and meta-analysis.

**Search Strategy:** Searching MEDLINE, Embase, Web of Science, SCOPUS, Cochrane Library, and clinical trial registries.

**Selection Criteria:** Randomized controlled trials of singleton gestations with a history of PTB and treated with a weekly intramuscular injection of 17-OHPC or placebo.

**Data Collection and Analysis:** A random meta-analysis model was performed for the PTB outcomes (<32, <35, and <37 weeks) and neonatal outcomes (neonatal death, grade 3 or 4 intraventricular hemorrhage, respiratory distress syndrome, bronchopulmonary dysplasia, necrotizing enterocolitis, and sepsis). Effect estimates were measured by relative risk ratio (RR) with a 95% confidence interval (CI).

**Main Results:** Six works were included. There were no statistically significant reductions in the PTB risk following the use of 17-OHPC at <32 weeks (RR = 0.61, 95% CI: 0.13–2.77, and *I*^2^ = 39%), <35weeks (RR = 0.60, 95% CI: 0.10–3.67, and *I*^2^ = 51%), and <37 weeks (RR = 0.68, 95% CI: 0.46–1, and *I*^2^ = 75%). Furthermore, all the neonatal outcomes were statistically similar between the two groups.

**Conclusion:** Treatment with 17-OHPC is not associated with reducing the risk of PTB or neonatal outcomes compared to placebo.

## Synopsis

Systematic review and meta-analyses of randomized trials showed that 17-OHPC is not associated with reducing the risk of PTB or neonatal outcomes compared to placebo.

## Introduction

The World Health Organization defined preterm birth (PTB) as delivery before 37 weeks of gestational age. The estimated global incidence of PTB was 15 million per year (1). Several risk factors were recognized for the recurrent PTB, including women with a history of prior PTB in any pregnancy and interpregnancy interval of fewer than 12 months (2).

The use of vaginal progesterone demonstrated a significant reduction in the risk of PTB (3), and also the use of 17-alpha-hydroxyprogesterone caproate (17-OHPC), which showed promising results (4). In 2011, the Food and Drug Administration (FDA) approved 17-OHPC for reducing the risk of PTB among pregnant women with a singleton pregnancy who have a history of singleton spontaneous PTB, and it was the only approved medication for this indication (5). However, in 2020, 17-OHPC (Makena) was recommended to be withdrawn from the market after failing the PROLONG trial to confirm the clinical benefit in reducing the risk of PTB and the benefit to neonates (6, 7).

Due to the inconsistence in the evidence between approval trial and PROLONG trial, this systematic review and meta-analysis aimed to determine the effect of 17-alpha-hydroxyprogesterone compared to placebo in reducing the risk of recurrent PTB and neonatal morbidity and mortality in women with singleton pregnancy and a history of at least one previous spontaneous PTB (SPTB).

## Methods

### Data Source and Searches

The research proposal for this work was registered in the International Prospective Register of Systematic Reviews (PROSPERO: CRD42021228756). The manuscript was prepared based on Preferred Reporting Items for Systematic Reviews (8). We conducted a comprehensive systematic search for the following databases: MEDLINE, Embase, Web of Science, SCOPUS, and The Cochrane Library, from inception to December 31, 2020 then updated till June 28, 2021. We also searched the following clinical trial registries: International Clinical Trials Registry Platform (ICTRP) https://apps.who.int/trialsearch/, The United States Clinical Trials Registry https://www.clinicaltrials.gov/, Australian New Zealand Clinical Trials Registry (ANZCTR) http://www.anzctr.org.au/TrialSearch.aspx, Clinical Trial Registry in Japan https://www.umin.ac.jp/ctr/, International Standard Randomized Controlled Trial Number Registry (ISRCTN registry) http://www.isrctn.com/, European Union Clinical Trials Register https://www.clinicaltrialsregister.eu/ctr-search/search, and Nederland's Trial Register https://www.trialregister.nl/. Moreover, we manually searched for any potential eligible works through the references of published reviews and selected works. The search strategies are available in the Supplementary Table 1.

### Eligibility Criteria

We included all randomized controlled trials that compared the weekly intramuscular injection of 17-OHPC (intervention group) to weekly intramuscular injection of placebo (control group) in singleton pregnant women with a prior history of SPTB. We excluded trials that involve twin or multiple pregnancies or those used exclusively in patients with preterm rupture of membrane history, HIV history, assisted reproductive technology, arrested PTB, successful tocolysis, and placenta praevia.

### Study Selection

After removing the duplicated and unrelated works, three investigators (AA, NF, and HA) independently reviewed the title and abstract, followed by a full-text review for works that met the inclusion criteria using the Rayyan software (9). Disagreement during the work selection was resolved by consensus.

### Data Extraction and Quality Assessment

All the three authors (AA, NF, and HA) independently extracted the basic characteristics of the included works then cross-checked them. Disagreement was resolved by consensus. The extracted data were recorded in an Excel spreadsheet and included primary author name, year of publication, work location, work period; key inclusion criteria; sample size used for both 17-OHPC group and placebo group; and the treatment duration. The outcome measures were recorded in an Excel spreadsheet and included birth outcomes at (<32, <35, and <37 weeks) of gestation and the neonatal outcomes: neonatal death, grade 3 or 4 intraventricular hemorrhage, respiratory distress syndrome, bronchopulmonary dysplasia, necrotizing enterocolitis, and sepsis. AA, NF, and HA extracted data independently, cross-checked, and utilized consensus to resolve any disagreement. The revised Cochrane risk-of-bias tool for randomized trials was used to assess the quality of the included works for any potential biases (10). The risk of bias was assessed independently by AA, NF, and HA, then cross-checked, and agreed consensually.

### Data Analysis

We performed a random effect meta-analysis model weighted by the Mantel–Haenszel method using Hartung and Knapp model for the outcomes reported in more than five works and DerSimonian and Laird random-effects model for outcomes reported in five works or less with a large ratio between the sizes of the works (11, 12). We reported the effect sizes of all meta-analyses as risk ratios (RR) with the corresponding 95% CI and graphed using forest plot figures. In addition, we quantified the between-work heterogeneity using the *I*^2^ statistics where *I*^2^ ≥ 75 percentage is indicative of high heterogeneity. We assessed the publication bias using Egger's test for the outcomes from four or more works. Subgroup analysis based on the location of the works [the United States (US vs. non-US)] was conducted for the outcome PTB at <32, <35, and <37 weeks. We also conducted sensitivity analyses for outcomes with substantial heterogeneity (≥75%) by removing one work at a time and analyzing the rest of the works to evaluate the impact of each work on the point of estimate and the heterogeneity. The statistical analysis was performed using R version 4.0.4.

## Results

### Studies' Characteristics

**Figure 1:**
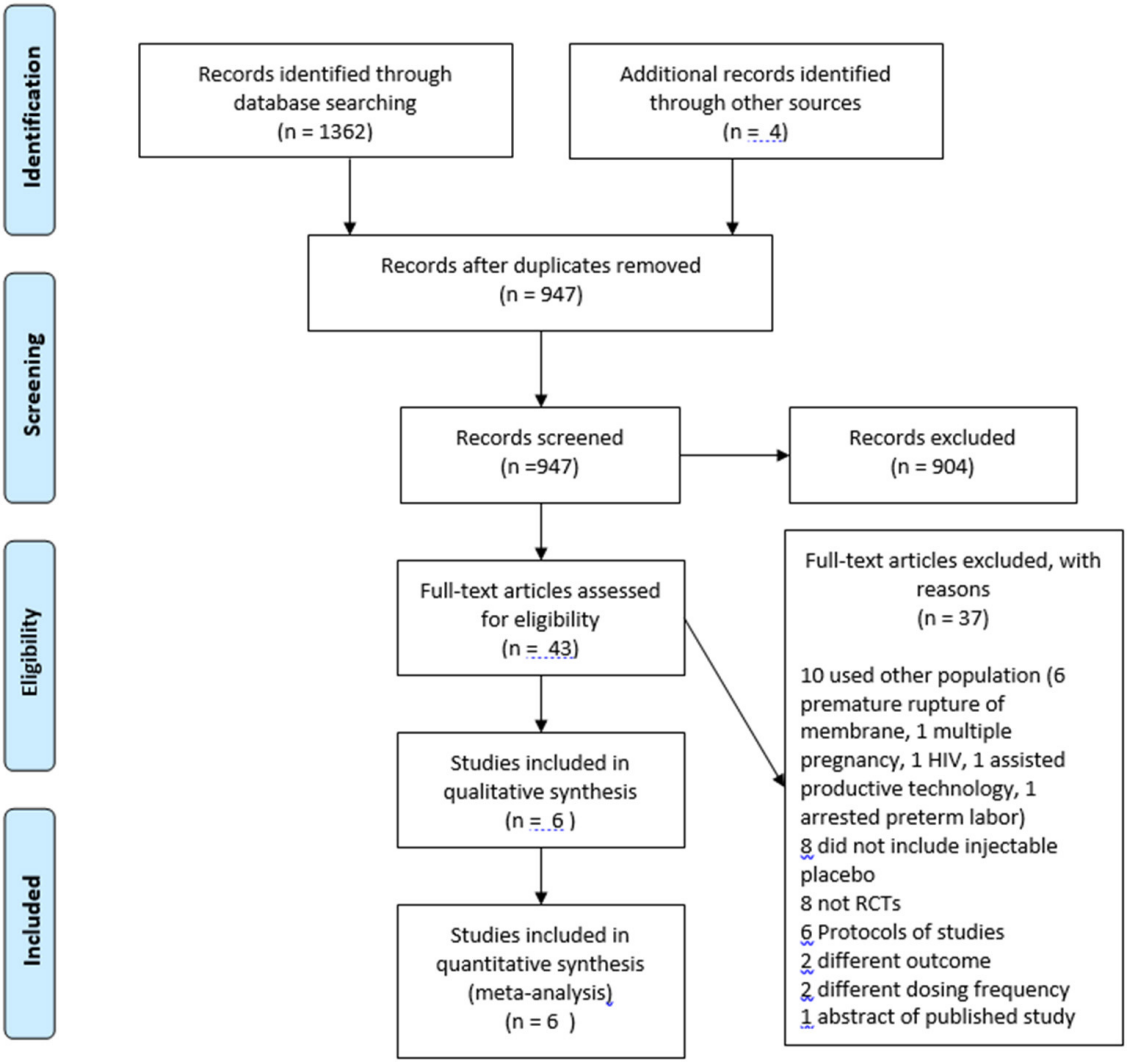
Flowchart of included and excluded works.

The databases search yielded 1,366 publications, and 43 publications were assessed using full-test for eligibility. Six randomized control trials met the inclusion and exclusion criteria (Figure 1) (4, 6, 13–16). The excluded works were summarized in Supplementary Table 2. The characteristics of the included works were summarized in Table 1. Four trials included 50 participants or less per arm (13–16). Three trials allowed cervical cerclage during the work period (6, 13, 14).

**Table 1:** Characteristics of the works included in the systematic review and meta-analysis.

**Study**	**Study type**	**Location**	**Study duration**	**Inclusion criteria**	**17P**	**Placebo**	**Treatment period**
					***N* = randomized (*n* = analyzed)**	***N* = randomized (*n* = analyzed)**	
Johnson et al. (13)	DBRT	USA, one center (Johns Hopkins Hospital)	NR	History of two spontaneous abortions or one PMD and one spontaneous abortion or 2 ≥ PMD, GA: <24 week	23 (18); 250 mg IM QW	27 (25), placebo IM QW	<24–36
Yemini et al. (14)	DBRCT	Israel	NR	History of at least two preterm deliveries or two spontaneous miscarriages or a combination of both	40 (39)	40 (40)	12–37th week
Meis et al. (4)	DBRCT	USA, 19 clinical centers	September 1999 to February 2002	SPTB history (singleton)	310 (306)	153 (153)	16–36
				GA: between 15^+0^ and 20^+3^ week			
Ibrahim et al. (10)	RCT	Egypt, one center (Ain Shams University maternity hospital, Cairo)	August 2006 to November, 2008	SPTB history (singleton)	25 (25)	25 (15)	Second trimester−36
				GA: second trimester			
Shahgheibi et al. (16)	DBRCT	Iran (Besat hospital in Sanandaj)	2013–2014	Singleton pregnancy with an accurate age of pregnancy; history of preterm labor before the 37th week; history of pre-mature birth; history of premature birth in sister; acquired and/or congenital uterine abnormalities; and age 18–45 years	50 (50)	50 (50)	24th−34th week
Blackwell et al. (6)	DBRT	93 clinical centers (41 in the United States and 52 outside the United States)	November 12, 2009, to October 8, 2018	Women 18 years old with a singleton pregnancy who had a documented previous pregnancy complicated by a singleton SPTB and who were 160/7 to 206/7 weeks in the current pregnancy	1,130 (1,130)	578 (578)	16–36

*PMD, premature delivery; DBRCT, double-blind, randomized controlled trial; RCT, randomized controlled trial; NR, not reported; SPTB, spontaneous PTB; GA, gestational age*.

### Risk of Bias Assessment

The overall risk of bias assessment of included works showed a low risk of bias in five works, but one work had some concern due to the open-label nature of the work and the lack of information about the baseline characteristics of included patients (Supplementary Table 3) (15). One work did not provide enough information about the adherence to the interventions which may raise a concern of the effect of adherence to the treatment (13). Another work had some concern due to the imbalance in the mean of previous preterm deliveries between the treatment arm and control arm at the baseline characteristics (4).

### Risk of PTB Before 32, 35, and 37 Weeks

**Figure 2:**
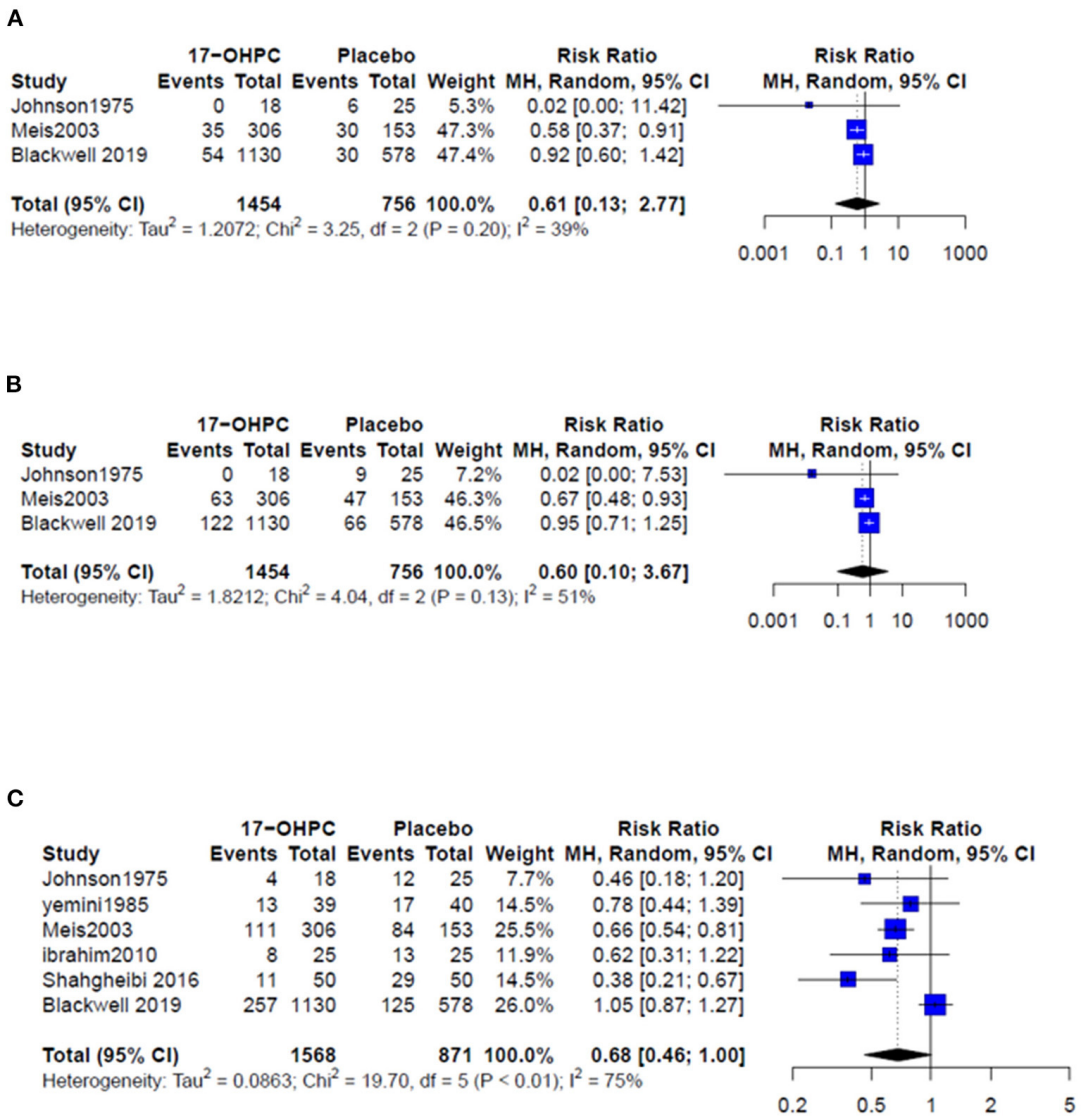
Forest plots of the risk of recurrent PTB below **(A)** 32 weeks, **(B)** 35 weeks, and **(C)** 37 weeks.

Three works provided data about the risk of PTB before 32 and 35 weeks (4, 6, 13). The pooled estimate showed no significant difference in reducing the risk of PTB prior 32 weeks (RR = 0.61, 95% CI: 0.13–2.77, and *I*^2^ = 39%, Figure 2A) and prior 35 weeks (RR = 0.60, 95% CI: 0.10–3.67, and *I*^2^ = 51%, Figure 2B). All six works provided data about the risk PTB before 37 weeks. The pooled estimate did not show a significant difference in the reduction in PTB risk before 37 weeks following the use of 17-OHPC compared to placebo (RR = 0.68, 95% CI: 0.46–1, and *I*^2^ = 75%, Figure 2C).

### Neonatal Outcomes

**Figure 3:**
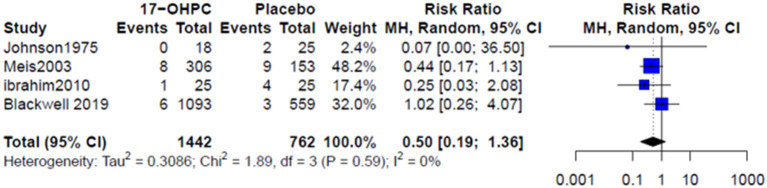
Forest plots of the risk of neonatal death.

**Figure 4:**
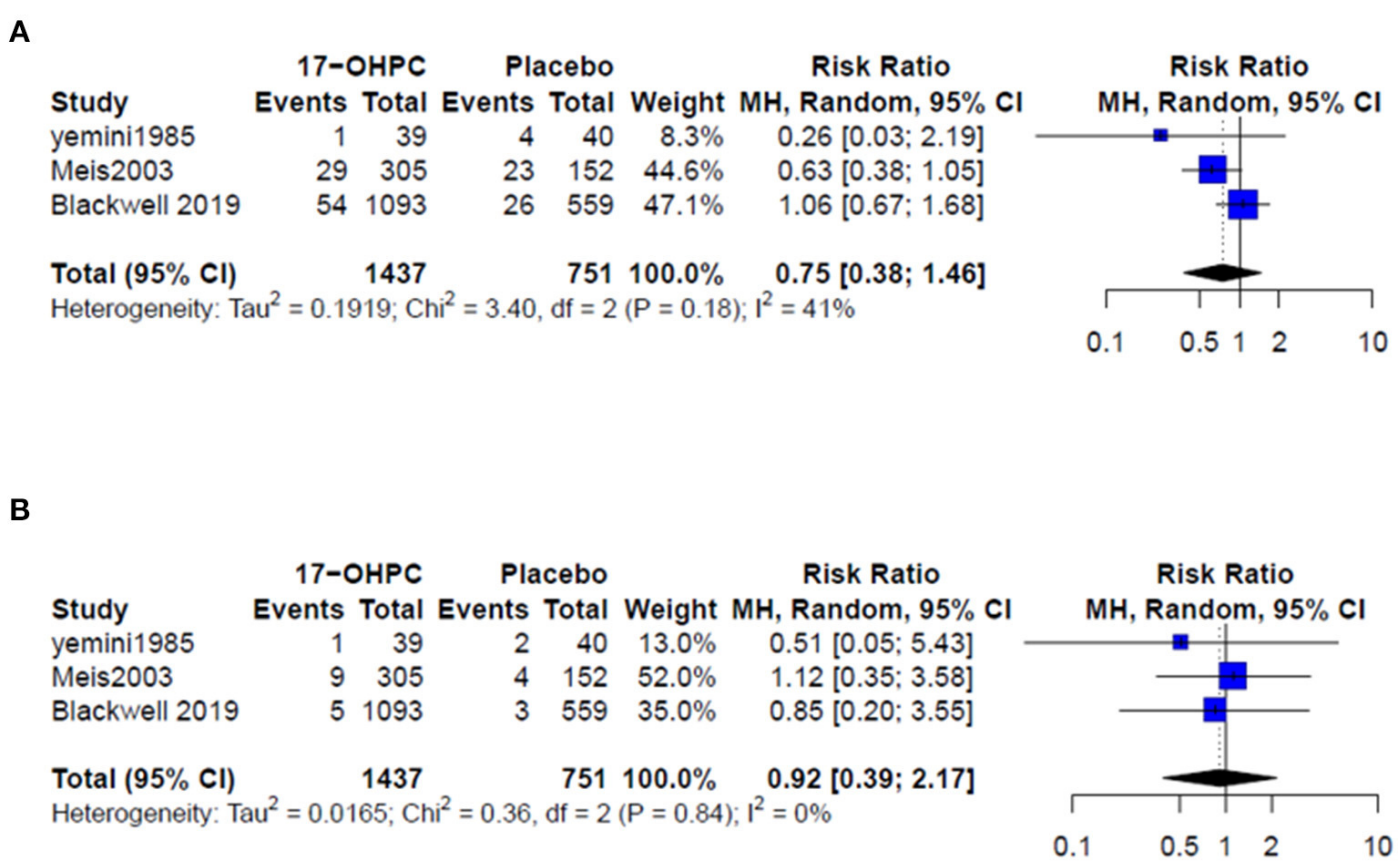
Forest plots of the risks of **(A)** respiratory distress and **(B)** sepsis.

**Figure 5:**
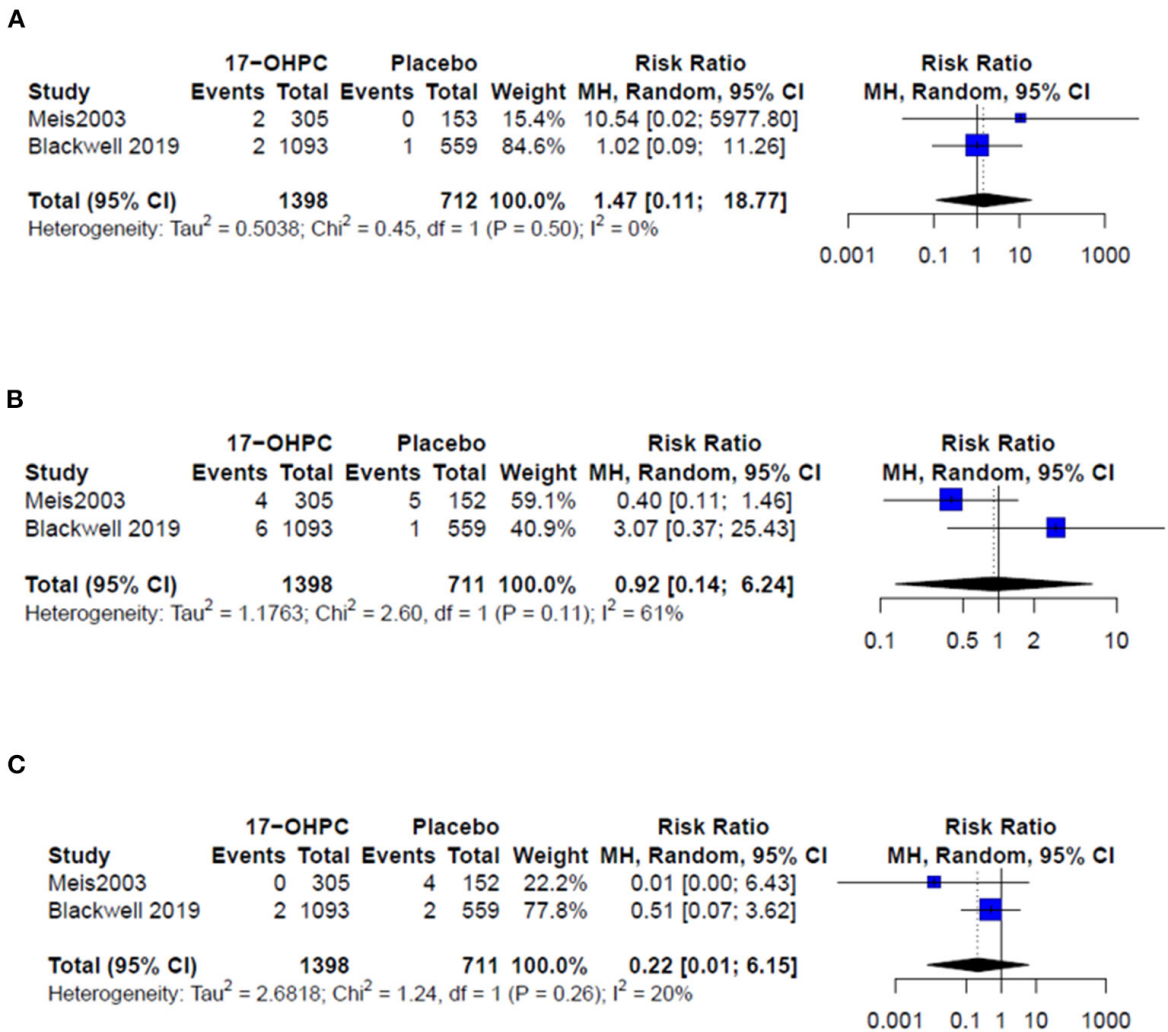
Forest plots of the risks of recurrent PTB below **(A)** grade 3 or 4 intravascular hemorrhage, **(B)** bronchopulmonary dysplasia, and **(C)** necrotizing enterocolitis.

Four works reported information about the risk of neonatal death (4, 6, 13, 15). There was no significant difference in reducing the risk of neonatal death between 17-OHPC and placebo (RR = 0.50, 95% CI: 0.19–1.36, and *I*^2^ = 0%, Figure 3). Three works had data about the risks of respiratory distress and sepsis (4, 6, 14). The pooled estimates did not show a significant difference in reducing the risk of both outcomes between 17-OHPC and placebo with (RR = 0.75, 95% CI: 0.38–1.46, and *I*^2^ = 41%, Figure 4A) for respiratory distress and (RR = 0.92, 95% CI: 0.39–2.17, and *I*^2^ = 0%, Figure 4B) for sepsis. Two works provided data about the risks of grade 3 or 4 intravascular hemorrhage, bronchopulmonary dysplasia, and necrotizing enterocolitis (4, 6). Results from the meta-analysis for these outcomes did not show a significant difference between 17-OHPC and placebo (Figures 5A–C).

### Subgroup Analyses

The results from subgroup analyses based on the region from three works for PTB <32 weeks and 35 weeks indicated that no significant difference between 17-OHPC and placebo in the US-based works with RR = 0.51, 95% CI: 0.07–3.90, and *I*^2^ = 0%, Supplementary Figure 1, and RR = 0.58, 95% CI: 0.03–12.92, and *I*^2^ = 18%, Supplementary Figure 2, respectively. However, the risk of PTB <32 weeks based on one work showed a 76% increase in the risk of PTB in 17-OHPC users vs. placebo users (RR = 1.76, CI: 1.02–3.02, Supplementary Figure 1). Data about the risk of PTB <37 weeks were obtained from three US-based works (4, 6, 13), and four non-US-based works (6, 14–16). The was no significant difference between 17-OHPC and placebo in the US-based works (RR = 0.78, 95% CI: 0.27–2.28, *I*^2^ = 80%, Supplementary Figure 3) and non-US-based works (RR = 0.69, 95% CI: 0.35–1.37, *I*^2^ = 72%, Supplementary Figure 3).

### Sensitivity Analyses

Exploring the effect of removing one work at a time for the risk of PTB <37 weeks (*I*^2^ = 75%) showed that removing PROLONG work resulted in reducing the heterogeneity (*I*^2^) to 0.04 % and changing the overall estimate to RR = 0.60, 95% CI: 0.43–0.83, which represent a significant reduction in the risk of PTB birth <37weeks following the use of 17-OHPC compared to placebo (Supplementary Figure 4).

### Publication Bias

The publication bias was assessed for two outcomes: PTB <37 weeks and neonatal death. Egger's test results for both outcomes did not suggest a significant publication bias with a *p*-value of 0.24 and 0.56, respectively.

## Discussion

Overall, the meta-analysis of the included works showed no significant association between 17-OHPC use and reduced risk for PTB in singleton gestations. They also found no significant differences in neonatal outcomes between 17-OHPC and placebo arms despite the low clinical and methodological heterogeneity across the works.

On the contrary, a recent meta-analysis (17) of four clinical trials comparing 17-OHPC vs. placebo or no treatment found that 17-OHPC can reduce PTB before 37 weeks of gestation by 29% (RR = 0.71; 95% CI = 0.53–0.96; *P* = 0.001). Regarding neonatal outcomes, a reduction in neonatal death by 68% (RR = 0.32; 95% CI = 0.15–0.66; *P* = 0.002) and a reduction in birthweights under 2,500 g by 35% (RR = 0.65; 95% CI = 0.50–0.84; *P* < 0.001) were reported. However, this meta-analysis did not include the data from the PROLONG trial. Compared to our review, which included only works that compared 17-OHPC and placebo treatments, Fernandez-Macias et al. meta-analysis compared 17-OHPC with placebo and no treatment groups (17).

Methodologically, both Meis et al. and PROLONG trials had almost similar inclusion and exclusion criteria (4, 6). However, there was an imbalance in baseline characteristics and risk factors in the treatment groups of PROLONG and Meis et al. trials. The PROLONG trial had a higher mean of gestational age, women's age, body mass index (BMI), smoking, alcohol, and illicit drug usage during pregnancy compared to the Meis et al. trial and a higher number of black women. The protocol of the PROLONG trial was also modified to allow the participation of women if they received any progesterone and stopped at least 4 weeks before the first dose of the work medication. However, PROLONG trial did not provide information about evaluating the results in patients who received any progesterone before the first dose of the work medication and also in patients who did not receive any progesterone. This would help to rule out any residual effect of using progesterone product before the randomization. The Meis et al. trial was conducted in the USA, while the PROLONG trial was a multinational, multicenter work, and the background rate of PTBs may not be similar in the included countries. In addition, the Meis et al. trial included patients from academic centers, which may not be a good representative of the general population.

Other works have also demonstrated the superiority of vaginal progesterone to intramuscular progesterone in reducing the risk for PTB. In a previous meta-analysis that included only randomized works comparing the efficacy of vaginal progesterone with that of 17-OHPC in preventing SPTB, lower rates of SPTB below 34 weeks were observed with vaginal progesterone (17.5 vs. 25.0%; RR = 0.71; 95% CI = 0.53–0.95) compared to 17-OHPC (18). The pooled analysis also found vaginal progesterone associated with lower rates of adverse maternal side effects and admission to NICU (18). Consistent with these findings, Maher et al., showed a beneficial effect of the vaginal progesterone over the intramuscular 17-OHPC in reducing the number of deliveries before 34 weeks of gestation (OR = 0.58; 95% CI = 0.37–0.89; *P* = 0.02), with lower adverse events in a Saudi-based cohort (19).

There are several strengths of our work. First, our analysis involved data from randomized controlled works of 2,451 women treated with either 17-OHPC or injectable placebo. Second, we included in our analysis the risk of preterm at three different time points (<32, <35, and <37 weeks) and also key neonatal outcomes. Third, we conducted a sensitivity analysis to assess the influence of removing one work at a time on the heterogeneity for the risk of recurrent PTB <37 weeks. The PROLONG work had a huge impact on the heterogeneity and the point estimate. It was the only multicenter, international trial with larger sample size. Finally, we were able to conduct a subgroup analysis to assess the difference in the risk of PTB (<32, <35, and <37 weeks) based on the work location to assess whether the effect of 17-OHPC differs in US-based works vs. non-US-based works. In general, the work location seems not to affect the overall conclusion even though one non-US-based work showed an increased risk of PTB <32 weeks among the 17-OHPC group, which may need more works to evaluate the effect of 17-OHPC on reducing the risk of PTB <32weeks.

We also acknowledge that our work has some limitations. First, not all outcomes were reported in the included works except in two works (4, 6). However, the Mesi et al. trial and PROLONG work were the largest trials which may allow us to perform an adequate estimate of the effect of 17-OHPC vs. placebo for all outcomes. Second, the included works did not provide enough information about the outcomes based on the cervical length at the starting of the work, smoking status, ethnicity, and the number of prior PTBs. Therefore, we were not able to run a meta-analysis to estimate the effect of 17-OHPC in these groups. Third, one of the works used an open-label method which may introduce some potential biases in managing and observing patients treated with 17-OHPC differed from those treated with placebo (15). However, in the sensitivity analysis, removing this work did not alter the conclusion (15). Finally, the lack of adequate information about the distribution of risk factors of PTB among treatment groups in all works may result in individual differences between the works. These differences may explain the heterogeneity between the works, especially for PTB outcomes <37 weeks.

In conclusion, our meta-analysis of the available randomized trials showed that the use of 17-OHPC might not be useful in reducing the risk of PTB or neonatal outcomes compared to placebo in women with singleton pregnancy and a history of SPTB. Further research discoveries are needed to provide effective treatment for PTB.

## Data Availability Statement

The original contributions presented in the study are included in the article/supplementary material. Further inquiries can be directed to the corresponding authors.

## Author Contributions

AA: analysis and interpretation of results. All authors: work conception, design and data collection, draft manuscript preparation, reviewed the results, and approved the final version of the manuscript.

## Author Disclaimer

The contents of this manuscript are solely the authors' views and may not be understood or quoted as being made on behalf of or reflecting the position of the Saudi Food and Drug Authority.

## Conflict of Interest

The authors declare that the research was conducted in the absence of any commercial or financial relationships that could be construed as a potential conflict of interest.

## Publisher's Note

All claims expressed in this article are solely those of the authors and do not necessarily represent those of their affiliated organizations, or those of the publisher, the editors and the reviewers. Any product that may be evaluated in this article, or claim that may be made by its manufacturer, is not guaranteed or endorsed by the publisher.
