# Supplementary material for: 17-Alpha-Hydroxyprogesterone vs. Placebo for Preventing of Recurrent Preterm Birth: A Systematic Review and Meta-Analysis of Randomized Trials
Source: Front Med (Lausanne). 2021 Dec 1;8:764855. doi: 10.3389/fmed.2021.764855 (PMC8671739; doi:10.3389/fmed.2021.764855)
Supplement: Supplementary file 1 [file Data_Sheet_1.docx]

**Supplemental Materials**

**Table S1: Search strategy**

| **1-PubMed**  ((("17-alpha-hydroxyprogesterone"[MeSH Terms] OR "17-alpha-hydroxyprogesterone"[All Fields] OR "17 hydroxyprogesterone"[All Fields]) AND ("premature birth"[MeSH Terms] OR ("premature"[All Fields] AND "birth"[All Fields]) OR "premature birth"[All Fields] OR ("preterm"[All Fields] AND "birth"[All Fields]) OR "preterm birth"[All Fields] OR “preterm delivery” "[All Fields] OR “preterm labor” "[All Fields]) AND singleton [All Fields] AND Placebo))) |
| --- |
| **2- SCOPUS**   ( ( 'hydroxyprogesterone  AND caproate'  OR  'hydroxyprogesterone'  OR  '17  AND alpha  AND hydroxyprogesterone'  OR  '17  AND alphahydroxyprogesterone'  OR  '17  AND hydroxyprogesterone' )  AND  ( 'prematurity'  OR  'preterm  AND birth'  OR  'premature  AND labor'  OR  'premature  AND birth'  OR  'preterm  AND labor'  OR  'preterm  AND delivery' )  AND  "placebo"  AND  ( 'singleton  AND pregnancy'  OR  'singleton' ) ) |
| **3- EMBASE**  **No.**  **Query**  **Results**  **229**  **#31**  **#15** AND **#22** AND **#27** AND **#29**  **229**  **#30**  **#15** AND **#22** AND **#27**  **1,103**  **#29**  **#15** AND **#22**  **42,554**  **#28**  **#23** OR **#24**  **474,854**  **#27**  **#25** OR **#26**  **474,854**  **#26**  **placebo**  **369,179**  **#25**  **'placebo'**/exp  **42,554**  **#24**  **singleton**  **185**  **#23**  **'singleton pregnancy'**/exp  **10,053**  **#22**  **#16** OR **#17** OR **#18** OR **#19** OR **#20** OR **#21**  **3,151**  **#21**  **'17 hydroxyprogesterone'**  **539**  **#20**  **'17 -alpha-hydroxyprogesterone'**  **539**  **#19**  **'17 -alpha hydroxyprogesterone'**  **555**  **#18**  **17** AND **'alpha hydroxyprogesterone'**  **1,775**  **#17**  **'hydroxyprogesterone caproate'**/exp  **7,504**  **#16**  **'hydroxyprogesterone'**/exp  **196,457**  **#15**  **#1** OR **#2** OR **#3** OR **#4** OR **#5** OR **#6** OR **#7** OR **#8** OR **#9** OR **#10** OR **#11** OR **#12** OR **#13** OR **#14**  **36,861**  **#14**  **preterm** AND **labor**  **34,582**  **#13**  **preterm** AND **delivery**  **15,905**  **#12**  **'preterm delivery'**  **9,241**  **#11**  **'preterm labor'**  **49,850**  **#10**  **'premature labor'**  **4,371**  **#9**  **'premature delivery'**  **36,364**  **#8**  **premature** AND **delivery**  **53,735**  **#7**  **premature** AND **labor**  **56,464**  **#6**  **premature** AND **birth**  **63,665**  **#5**  **preterm** AND **birth**  **119,510**  **#4**  **'premature birth'**/exp  **49,021**  **#3**  **'premature labor'**/exp  **27,045**  **#2**  **'preterm birth'**  **119,510**  **#1**  **'prematurity'**/exp |

**Table S2: Excluded studies after full-text assessment**

|  | **Study** | **Reason** |
| --- | --- | --- |
| 1 | Combs, C. Andrew, et al. "17-Hydroxyprogesterone caproate to prolong pregnancy after preterm rupture of the membranes: early termination of a double-blind, randomized clinical trial." BMC research notes 4.1 (2011): 1-9. | used other population ( premature rupture of membrane) |
| 2 | Langen, Elizabeth, et al. "291: A double-blind, randomized, placebo controlled trial of 17 alpha-hydroxy-progesterone caproate (17-OHP) in the management of preterm, premature rupture of membranes." American Journal of Obstetrics & Gynecology 214.1 (2016): S167. | used other population ( premature rupture of membrane) |
| 3 | ClinicalTrials.gov [Internet]. Bethesda (MD): National Library of Medicine (US). Identifier: NCT01119963, Progesterone (17P, Makena®) for Prolongation of Pregnancy in Women With Preterm Rupture of the Membranes (PROM) (17PinPROM), https://clinicaltrials.gov/ct2/show/NCT01119963 | used other population ( premature rupture of membrane) |
| 4 | Mirzaei, Fatemeh, and Parvin Moradi. "Effects of progesterone on latency period in patients with preterm premature rupture of membranes during 24-34 weeks of pregnancy." Journal of Kerman University of Medical Sciences 22.3 (2015): 240-248. | used other population ( premature rupture of membrane) |
| 5 | Combs, C. Andrew, et al. "17-hydroxyprogesterone caproate for preterm rupture of the membranes: a multicenter, randomized, double-blind, placebo-controlled trial." American journal of obstetrics and gynecology 213.3 (2015): 364-e1. | used other population ( premature rupture of membrane) |
| 6 | Briery, Christian M., et al. "Women with preterm premature rupture of the membranes do not benefit from weekly progesterone." American journal of obstetrics and gynecology 204.1 (2011): 54-e1. | used other population ( premature rupture of membrane) |
| 7 | Lim, Arianne C., et al. "17α-hydroxyprogesterone caproate for the prevention of adverse neonatal outcome in multiple pregnancies: a randomized controlled trial." Obstetrics & Gynecology 118.3 (2011): 513-520. | used other population (multiple pregnancy) |
| 8 | Price, Joan T., et al. "Intramuscular 17-hydroxyprogesterone caproate to prevent preterm birth among HIV-infected women in Zambia: study protocol of the IPOP randomized trial." BMC pregnancy and childbirth 19.1 (2019): 1-9. | used other population (HIV) |
| 9 | Aflatoonian, Abbas, Hoora Amouzegar, and Razieh Dehghani Firouzabadi. "Efficacy of 17α-hydroxy progestrone on decreasing preterm labor in ART pregnancies: A randomized clinical trial." International Journal of Reproductive BioMedicine 11.10 (2013): 785-0. | used other population (Assisted  reproductive technology) |
| 10 | Briery, Christian M., et al. "The use of 17-hydroxy progesterone in women with arrested preterm labor: a randomized clinical trial." The Journal of Maternal-Fetal & Neonatal Medicine 27.18 (2014): 1892-1896. | used other population (arrested preterm labor) |
| 11 | Winer, Norbert, et al. "17 alpha-hydroxyprogesterone caproate does not prolong pregnancy or reduce the rate of preterm birth in women at high risk for preterm delivery and a short cervix: a randomized controlled trial." American journal of obstetrics and gynecology 212.4 (2015): 485-e1. | did not include injectable placebo |
| 12 | Jafarpour, Hamed, et al. "Effect of 17 α-Hydroxyprogesterone Caproate on the Prevention of Preterm Labor: A Randomized Controlled Trial Study." Journal of Midwifery and Reproductive Health 8.3 (2020): 2317-2323. | did not include injectable placebo |
| 13 | Saghafi, Nafiseh, et al. "Efficacy of 17α‐hydroxyprogesterone caproate in prevention of preterm delivery." Journal of Obstetrics and Gynaecology Research 37.10 (2011): 1342-1345. | did not include injectable placebo |
| 14 | Rozenberg, Patrick, et al. "Prevention of preterm delivery after successful tocolysis in preterm labor by 17 alpha-hydroxyprogesterone caproate: a randomized controlled trial." American journal of obstetrics and gynecology 206.3 (2012): 206-e1. | did not include injectable placebo |
| 15 | Shaamash, Ayman H., Mohammed K. Ali, and Khalid M. Attyia. "Intramuscular 17α-hydroxyprogesterone caproate to decrease preterm delivery in women with placenta praevia: a randomised controlled trial." Journal of Obstetrics and Gynaecology 40.5 (2020): 633-638. | did not include injectable placebo |
| 16 | Winer, Norbert, et al. "4: Prevention of preterm delivery by 17 alpha-hydroxyprogesterone caproate in high risk asymptomatic singleton gestations with a short cervix: a randomized controlled trial." American Journal of Obstetrics & Gynecology 210.1 (2014): S3. | did not include injectable placebo |
| 17 | Facchinetti, Fabio, et al. "Progestogens for maintenance tocolysis in women with a short cervix: a randomized controlled trial." Obstetrics & Gynecology 130.1 (2017): 64-70. | did not include injectable placebo |
| 18 | Ndoni, E., et al. "Treatment with different types of progesterone in prevention of preterm delivery." J Matern Neonatal Med 23.suppl (2010): 305. | did not include injectable placebo |
| 19 | Deeks, Emma D. "17 α-Hydroxyprogesterone Caproate (Makena™)." Pediatric Drugs 13.5 (2011): 337-345. | not RCTs |
| 20 | Meis, Paul J. "17 Hydroxyprogesterone for the prevention of preterm delivery." Obstetrics & Gynecology 105.5 (2005): 1128-1135. | not RCTs |
| 21 | O'Brien, John M. "17α-Hydroxyprogesterone Caproate for the Prevention of Adverse Neonatal Outcome in Multiple Pregnancies: A Randomized Controlled Trial." Obstetrics & Gynecology 119.2 (2012): 384-385. | not RCTs |
| 22 | Petrini, Joann R., et al. "Estimated effect of 17 alpha-hydroxyprogesterone caproate on preterm birth in the United States." Obstetrics & Gynecology 105.2 (2005): 267-272. | not RCTs |
| 23 | Brancazio, Leo, et al. “Prevention of Recurrent Preterm Delivery by 17 Alpha-Hydroxyprogesterone Caproate.” N Engl J Med, vol. 349, no. 11, 2003, pp. 1087–88 | not RCTs |
| 24 | Meis, Paul J. "The role of 17 α-hydroxyprogesterone caproate in the prevention of preterm birth." Women’s health 2.6 (2006): 819-824. | not RCTs |
| 25 | Legardeur, H., L. Mandelbrot, and G. Kayem. "What use of progesterone to prevent preterm birth?." Gynecologie, obstetrique & fertilite 41.7-8 (2013): 459-464. | not RCTs |
| 26 | Koontz, Gretchen. "Does gestational age at randomization affect the efficacy of alpha hydroxyprogesterone caproate (17-OHCP) in preventing recurrent preterm delivery?." American Journal of Obstetrics and Gynecology 6.193 (2005): S55. | not RCTs |
| 27 | ClinicalTrials.gov [Internet]. Bethesda (MD): National Library of Medicine (US). Identifier: NCT01004029, Confirmatory Study of 17P vs Vehicle for Prevention of Preterm Birth in Women w/ Previous Spontaneous Preterm Delivery (PROLONG) <https://clinicaltrials.gov/ct2/show/NCT01004029> | Protocols of studies |
| 28 | ClinicalTrials.gov [Internet]. Bethesda (MD): National Library of Medicine (US). Identifier: NCT03297216, Improving Pregnancy Outcomes With Progesterone (IPOP)  <https://clinicaltrials.gov/ct2/show/NCT03297216> | Protocols of studies |
| 29 | International Clinical Trials Registry, World Health Organization (WHO), A Phase 3B, Multi-Center, Randomized, Double-Blind Study of Hydroxyprogesterone Caproate Injection, 250 mg/mL, Versus Vehicle for the Prevention of Preterm Birth in Women with a Previous Singleton Spontaneous Preterm Delivery  <http://www.who.int/trialsearch/Trial2.aspx?TrialID=EUCTR2009-014696-52-IT> | Protocols of studies |
| 30 | Lim, Arianne C., et al. "Progesterone for the prevention of preterm birth in women with multiple pregnancies: the AMPHIA trial." BMC pregnancy and childbirth 7.1 (2007): 1-6. | Protocols of studies |
| 31 | Blackwell, Sean C., et al. "PROLONG clinical study protocol: hydroxyprogesterone caproate to reduce recurrent preterm birth." American journal of perinatology 35.12 (2018): 1228-1234. | Protocols of studies |
| 32 | ClinicalTrials.gov [Internet]. Bethesda (MD): National Library of Medicine (US). Identifier: NCT00120640, Treatment of Preterm Labor With 17 Alpha-hydroxyprogesterone Caproate  https://clinicaltrials.gov/ct2/show/NCT00120640 | Protocols of studies |
| 33 | Facchinetti, Fabio, et al. "17α-hydroxy-progesterone effects on cervical proinflammatory agents in women at risk for preterm delivery." American Journal of Perinatology 25.08 (2008): 503-506. | different outcome |
| 34 | Northe, Allison. “Determinants of Compliance in a Randomized Trial in High Risk Pregnancy.” Clinical Trials, vol. 7, no. 4, 2010, p. 454 | different outcome |
| 35 | Ndoni, E., et al. "Treatment with different types of progesterone in prevention of preterm delivery." J Matern Neonatal Med 23.suppl (2010): 305. | different dosing frequency |
| 36 | Tan, Peng Chiong, et al. "Single dose 17 alpha-hydroxyprogesterone caproate in preterm labor: a randomized trial." Archives of gynecology and obstetrics 285.3 (2012): 585-590. | different dosing frequency |
| 37 | Johnson, John, et al. “The Efficacy of 17α Hydroxyprogesterone Caproate in Preventing Premature Labor.” GYNEC.INVEST., vol. 5, no. 1, 1974 | abstract of published study |

**Table S3: Risk of bias assessment**

| **Study** | **D1** | **D2(A)** | **D2(B)** | **D3** | **D4** | **D5** | **Overall** |
| --- | --- | --- | --- | --- | --- | --- | --- |
| Johnson1975 | Low | Low | Some concerns | Low | Low | Low | Low |
| Yemini1985 | Low | Low | Low | Low | Low | Low | Low |
| Meis2003 | Some concerns | Low | Low | Low | Low | Low | Low |
| ibrahim2010 | Some concerns | Some concerns | Some concerns | Low | Some concerns | Low | Some concerns |
| Shahgheibi 2016 | Low | Low | Low | Low | Low | Low | Low |
| Blackwell 2019 | Low | Low | Low | Low | Low | Low | Low |

Domain 1(D1): Risk of bias arising from the randomization process

Domain 2 A (D2A): Risk of bias due to deviations from the intended interventions (effect of assignment to intervention)

Domain 2 B (D2B): Risk of bias due to deviations from the intended interventions (effect of adhering to intervention)

Domain 3 (D3): Missing outcome data

Domain 4 (D4): Risk of bias in measurement of the outcome

Domain 5 (D5): Risk of bias in selection of the reported result

**Subgroup Analysis based on study location**


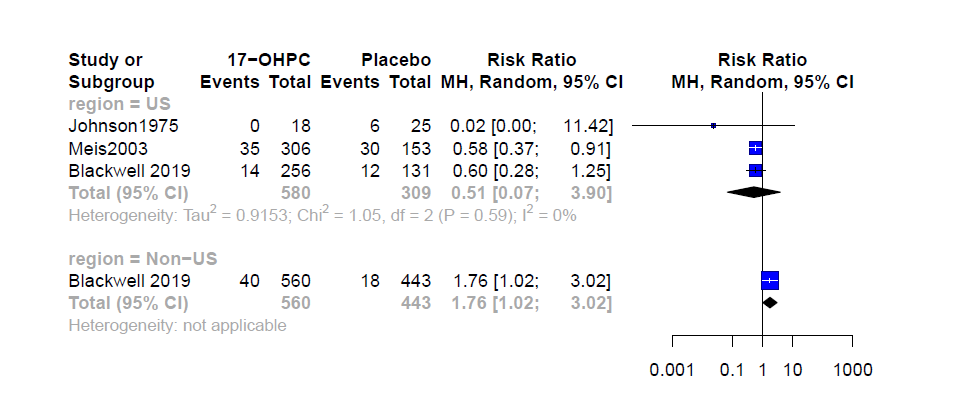


**Figure S1:** Forest plots of the risk of recurrent preterm birth bellow 32 weeks


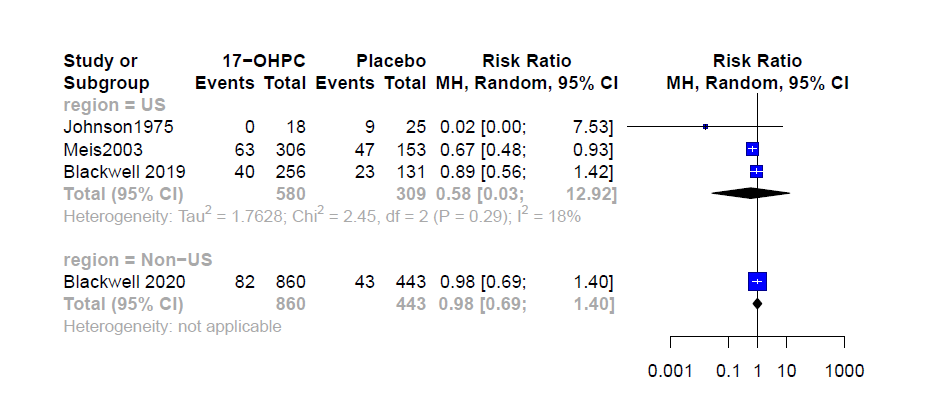


**Figure S2:** Forest plots of the risk of recurrent preterm birth bellow 35 weeks


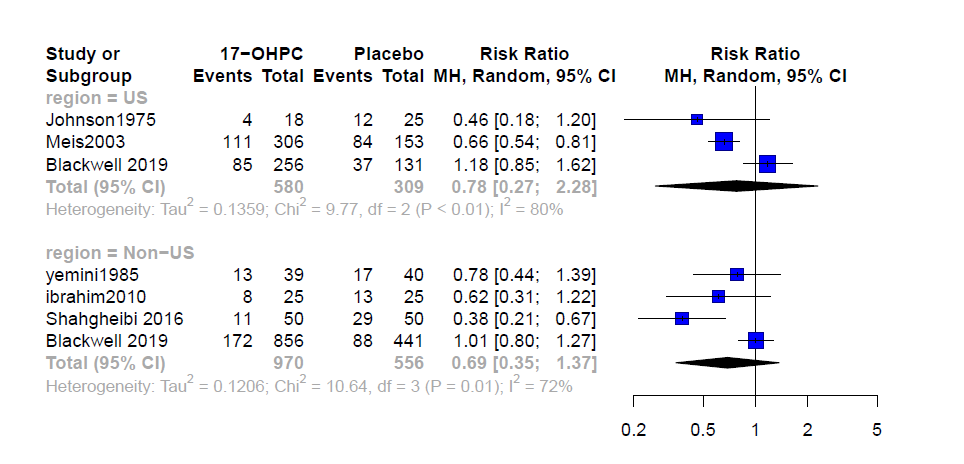


**Figure S3:** Forest plots of the risk of recurrent preterm birth bellow 37 weeks

**Sensitivity Analyses:**


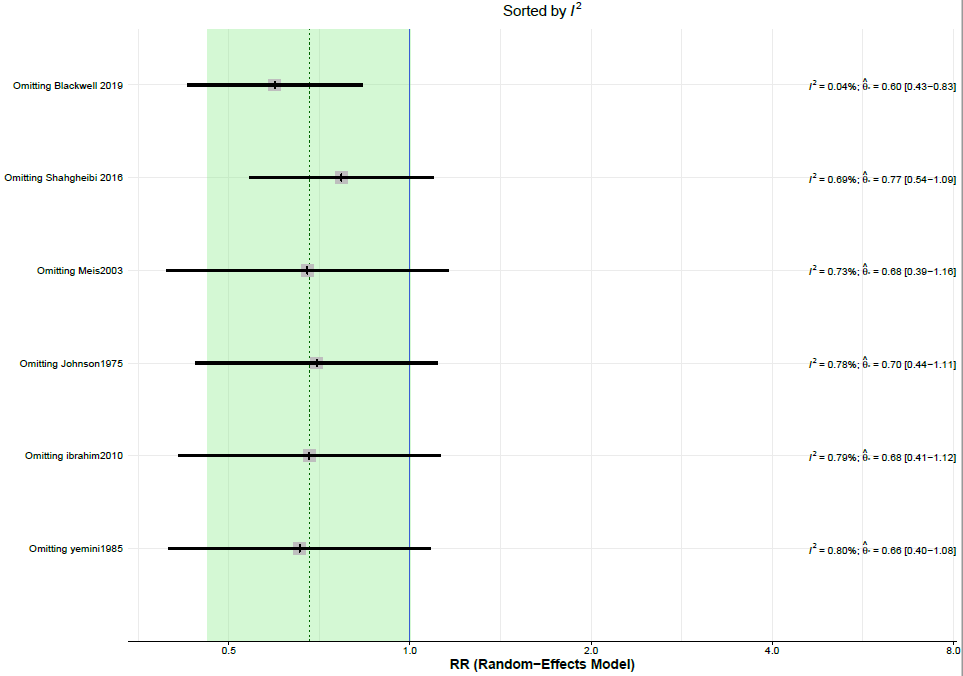


**Figure S4:** Effect of removing one study at a time on the heterogeneity for the risk of preterm < 37 weeks
